# *BRCA1* germline mutation and glioblastoma development: report of cases

**DOI:** 10.1186/s12885-015-1205-1

**Published:** 2015-03-26

**Authors:** Meriem Boukerroucha, Claire Josse, Karin Segers, Sonia El-Guendi, Pierre Frères, Guy Jerusalem, Vincent Bours

**Affiliations:** 1 University of Liège, GIGA-Cancer Research, Human Genetics Unit, Liège, Belgium; 2 Division of Medical Oncology, Liège University and CHU Sart Tilman Liège, Liège, Belgium; 3 Human Genetics Department, Liège University Hospital, Liège, Belgium

**Keywords:** *BRCA1*, Glioblastoma, Breast cancer

## Abstract

**Background:**

Germline mutations in breast cancer susceptibility gene 1 (*BRCA1*) increase the risk of breast and ovarian cancers. However, no association between *BRCA1* germline mutation and glioblastoma malignancy has ever been highlighted.

Here we report two cases of *BRCA1* mutated patients who developed a glioblastoma multiform (GBM).

**Cases presentation:**

Two patients diagnosed with triple negative breast cancer (TNBC) were screened for *BRCA1* germline mutation. They both carried a pathogenic mutation introducing a premature STOP codon in the exon 11 of the *BRCA1* gene. Few years later, both patients developed a glioblastoma and a second breast cancer. In an attempt to clarify the role played by a mutated *BRCA1* allele in the GBM development, we investigated the *BRCA1* mRNA and protein expression in breast and glioblastoma tumours for both patients. The promoter methylation status of this gene was also tested by methylation specific PCR as *BRCA1* expression is also known to be lost by this mechanism in some sporadic breast cancers.

**Conclusion:**

Our data show that *BRCA1* expression is maintained in glioblastoma at the protein and the mRNA levels, suggesting that loss of heterozygosity (LOH) did not occur in these cases. The protein expression is tenfold higher in the glioblastoma of patient 1 than in her first breast carcinoma, and twice higher in patient 2. In agreement with the high protein expression level in the GBM, *BRCA1* promoter methylation was not observed in these tumours.

In these two cases, despite of a *BRCA1* pathogenic germline mutation, the tumour-suppressor protein expression is maintained in GBM, suggesting that the *BRCA1* mutation is not instrumental for the GBM development.

## Background

Breast cancer susceptibility gene 1 (*BRCA1*) is the first tumour suppressor gene identified in familial breast cancer. Located on chromosome 17 (17q21), this gene encodes a multifunctional protein involved in several cellular processes such as DNA repair, chromatin remodelling and cell cycle regulation [1]. Several studies reported that germline mutations in *BRCA1* gene increase the risk to develop breast and ovarian cancers [1,2]. Indeed, women bearing pathogenic germline *BRCA1* mutations have a 45% to 80% risk to develop breast cancer by age 70, and 36% to 66% for ovarian cancer [3]. *BRCA1* somatic mutations are very rare but its promoter methylation is reported to occur in about 7% to 30% of breast and ovarian sporadic cancers [4,5]. In glioma, genome-wide association studies have identified common genetic variations in 7 genes that increase glioma risk (*TERT, EGFR, CCDC26, CDKN2A, CDKN2B, PHLDB1* and *RTEL1*) but *BRCA1* is not among them and there is no known association between *BRCA1* gene and glioblastoma multiforme (GBM) [6-8]. Indeed, Elmariah and co-authors reported the case of a patient mutated for *BRCA1* who developed glioblastoma but they did not investigate *BRCA1* mRNA and protein expression in the GBM [9]. Piccirilli and co-authors have also studied 11 cases of GBM occurring after mammary carcinoma, but their genomic status concerning *BRCA1* was unknown [10].

Here, we report two cases of patients with *BRCA1* germline mutation treated for breast cancer who developed glioblastoma few years after breast cancer diagnosis. The first patient had a triple negative breast cancer (TNBC) and six years later, a glioblastoma multiforme. In a very similar pattern, the second patient developed also a triple negative breast cancer and five years later a glioblastoma. In an attempt to clarify the role played by a mutated *BRCA1* allele in the GBM development, we assessed *BRCA1* mRNA and protein expression in the two tumour types for each patient. We also checked the *BRCA1* promoter methylation status.

Ethical approval was obtained from the local institutional ethical board (Comité d’éthique hospitalo-facultaire universitaire de Liège) in compliance with the Helsinki declaration, with the approval file number n°2010/229.

## Cases presentation

### Patient 1

A 28-years-old woman was diagnosed in 2000 with a ductal carcinoma of the left breast.

After radical mastectomy, histologic analysis revealed a 20 mm tumour with an infiltrating ductal carcinoma. Immunologic analysis demonstrated no expression of oestrogen and progesterone receptors (ER- and PR-) and no overexpression of HER2.

The patient was staged as T1N0M0 stage IA and received FEC adjuvant chemotherapy (FEC: Fluorouracil, Epirubicin, Cyclophosphamide). The proliferation marker Ki67 was expressed by 50% of the tumour cells.

Six years later, the patient developed a glioblastoma. After complete macroscopic surgical resection, the tumour was characterized as stage IV according to the WHO classification. The proliferative marker Ki67 was expressed by 40% of the tumour cells. The patient received temozolomide chemotherapy and radiotherapy followed by chemotherapy alone (6 cycles).

Two years after her diagnosis of GBM, she developed a carcinoma in the right breast. After mastectomy, the histologic and immunologic analysis of the 26 mm tumour revealed an infiltrating ductal carcinoma, negative for oestrogen and progesterone receptors but with HER2 gene amplification. The patient was staged as T2N0M0 stage IIA and received adjuvant chemotherapy targeted therapy (docetaxel and trastuzumab) for one year.

She died in 2012 after two relapses of the GBM.

**Figure 1:**
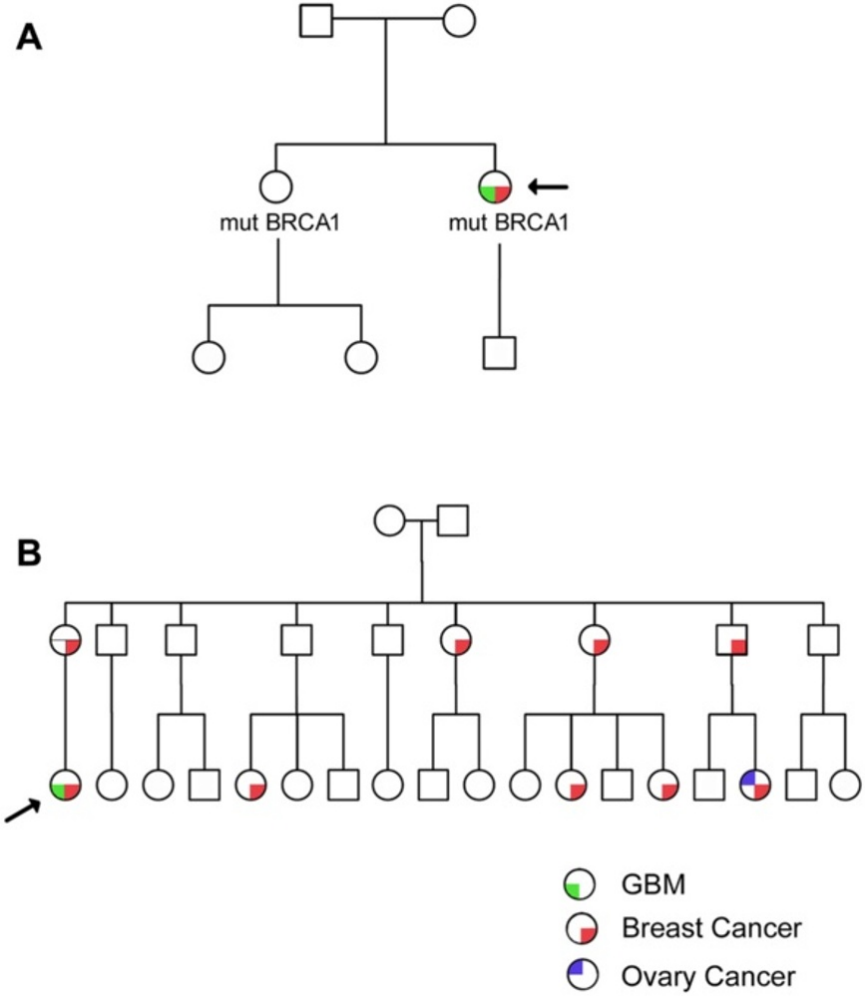
**Family trees of the patients. A**. Patient 1(arrow) **B**. Patient 2 (arrow). Cancer affected individuals are indicated.

*BRCA1* genetic testing was performed after the first breast cancer. The family tree is represented in Figure 1.A.

### Patient 2

A 56–years-old woman was diagnosed in 2005 with a ductal carcinoma in the left breast. After mastectomy, histological analysis revealed a 20 mm tumour with an infiltrating ductal carcinoma. Immunologic analysis demonstrated absence of hormone receptors expression (ER-, PR-) as well as absence of HER2 overexpression. Lymph nodes were not infiltrated. The tumour was classified as T2N0Mx stage IIA. The patient was treated with 6 cures of FEC chemotherapy. She developed metastases at the level of cervical and dorsal vertebra and received palliative chemotherapy (paclitaxel) and zoledronic acid.

Considering the family history of the patient (Figure 1B) and after molecular analysis of *BRCA1* gene, the patient was subjected to oophorectomy and hysterectomy.

Five years later, the patient developed a second breast carcinoma in the right breast. After mastectomy, histologic analysis revealed an 40 mm *in situ* ductal carcinoma associated with a 2 mm infiltrating ductal carcinoma and the absence of sentinel lymph node infiltration. Immunologic analysis of the invasive carcinoma demonstrated the absence of hormone receptors expression and an absence of HER2 protein overexpression. The invasive tumour was classified as pT1aN0Mx with a proliferative index based on Ki67 expression of 35%.

Few days after tumour resection, the patient presented mental confusion and a brain scan showed a mass at the fronto-insular level. After surgical excision, the histologic analysis revealed a glioblastoma characterized as stage IV according to the WHO classification. The patient received temozolomide chemotherapy and radiotherapy. One year later, the patient had surgical resection of progressive glioblastoma.

She died in 2011.

### Molecular analysis

#### DNA isolation

Tumour determined by a pathologist was manually macro-dissected from FFPE tissues. DNA was isolated from the first triple negative breast carcinoma and from the GBM in the two patients.

Blood samples were also collected in both patients to establish the *BRCA1* genomic status, after genetic counselling. Genomic DNA from leucocytes was extracted by standard phenol procedure.

#### BRCA1 gene analysis

All coding exons of *BRCA1* gene were subjected to PCR amplification. Amplicons were denatured, heteroduplexed and evaluated for the presence of mutations by Denaturing High Performance Liquid Chromatography (DHPLC) using product-specific melting and solvent conditions. All amplicons showing abnormal DHPLC pattern were sequenced by Sanger sequencing using ABI 3130 and following manufacturer recommendations. The reference sequence *BRCA1* (NM_007294.3) has been used for HGVS-approved amino acid numbering.

The following pathogenic mutations were founded by this method:

Patient 1: c.3481_3491del (p.Glu1161Phefs*3). This deletion of 11 bp leads to a frame-shift and premature Stop codon. This mutation has been previously identified in our population.

Patient 2: c.2722G > T (p.Glu908*). This mutation induces a premature Stop codon and is frequently identified in our Belgian population (11% of our *BRCA1* mutations).

These mutations were confirmed on independent samples by direct Sanger sequencing.

Moreover, the screening for large genomic rearrangement was performed using Multiplex ligation dependent probe amplification MLPA kits P002-C2 (MRC Holland) on the *BRCA1* gene region. This experiment did not highlight any abnormal genomic copy number change.

In attempt to identify LOH, screening for the *BRCA1* mutation has been performed by Sanger sequencing on DNA extracted from cerebral tumours of the two patients. However, the DNA quality obtained from GBM of patient 1 did not allow the amplification of the target region and the subsequent *BRCA1* sequencing. Regarding patient 2, the *BRCA1* mutation was identified in heterozygous status, suggesting that no loss of heterozygosity occurred.

#### BRCA1 and O^6^-methylguanine-DNA methyltransferase (MGMT) methylation status

The methylation status of *BRCA1* and *MGMT* promoter was assessed by methylation specific polymerase chain reaction (MSP-PCR) as previously described [11,12].

DNA extracted from tumours was first treated by sodium bisulfite using the EZ DNA Methylation Kit (Zymo Research) following manufacturer recommendations. MSP-PCR was done following the protocol of Esteller and co-authors [13].

**Figure 2:**
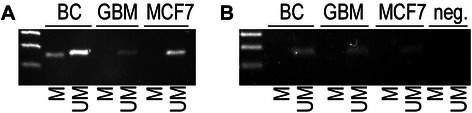
**Methylation status of*BRCA1*promoter in tumours. A** Patient 1. **B** Patient 2. M = methylated, UM = unmethylated, BC = breast cancer tissue, GBM = glioblastoma tissue, MCF7 = MCF-7 breast cancer cell line known to have unmethylated *BRCA1* promoter.

We assessed the methylation status of *BRCA1* promoter in the TNBC and GBM tumours for the two patients. A methylation was observed in TNBC tumour but not in GBM tumour for patient 1 as shown in Figure 2A. Regarding patient 2, the *BRCA1* promoter was unmethylated in both tumours (Figure 2B).

On the other hand, the *MGMT* promoter was methylated in GBM of both patients (Data not shown).

#### BRCA1 mRNA expression

The mRNA expression was assessed by *in situ* hybridization using RNA scope technology (ACD) for FFPE samples. In this experiment, the target probes are designed as double-Z as described by Fay Wang and co-authors [14]. The *BRCA1* probe is complementary to the coding region aa369-1482, spanning exon 5 to exon 11. To quantify the expression, spots and cells were independently and blindly counted twice, in subzones of the tumour. The ratio between the number of spots and the number of cells was compared to those of a positive control (MCF-7 cell line).

The mRNA expression level was 17,7% in breast carcinoma and 8% in GBM of patient 1.

**Figure 3:**
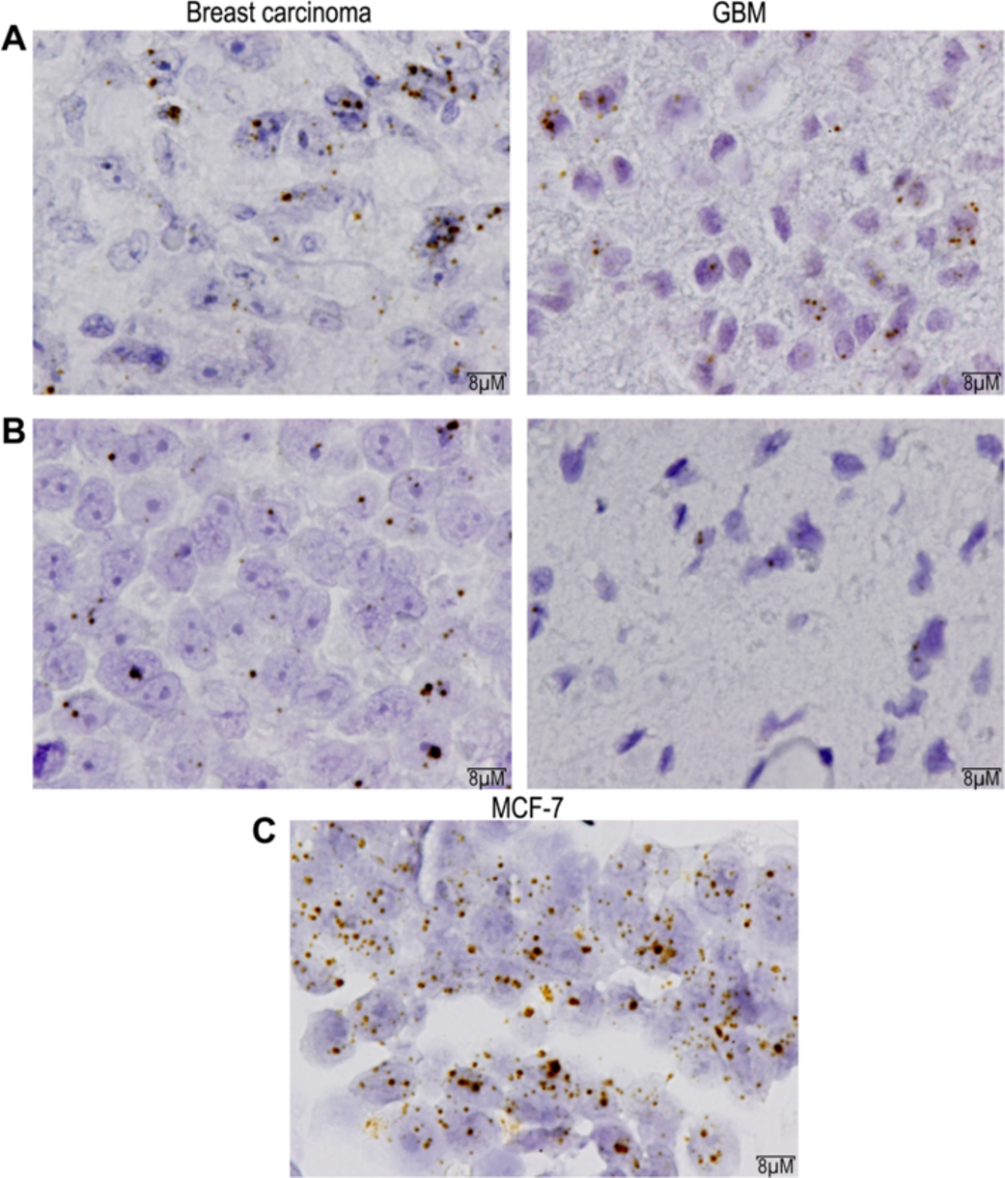
***BRCA1*mRNA expression by*in situ*hybridization. A**. Patient 1. Left : breast tumour; Right : GBM. **B**. Patient 2. Left : breast tumour; Right : GBM. **C**. Positive control : MCF7 cells.

Regarding patient 2, the mRNA expression level was 10,24% in breast tumour and 12,67% in GBM tumour. Representative images of the *BRCA1* mRNA expression in breast tumour and in GBM of both patients are represented in Figure 3.

#### BRCA1 protein expression

The protein level expression was assessed by proximity ligation assay (Duolink in situ detection reagents – Sigma). This assay is more specific than the conventional immunohistochemistry thanks to the use of two primary antibodies directed against two epitopes of the same protein. The primary antibodies are raised in different species and are recognized by two secondary antibodies coupled with oligonucleotide probes. After ligation of the two probes, the circular DNA is amplified by polymerase reaction. The detection is performed using horse radish peroxidase (HRP) labelled probes and a chromogenic reaction using 3,3’Diaminobenzidine (DAB). In order to generate a signal only with the full length *BRCA1* protein, the two primary antibodies where chosen to be specific to the N-term ([MS110] ab16780, Abcam) and C-term domain (Sigma, SAB4502848) of the *BRCA1* protein, respectively.

The protein expression level was estimated using the same quantification method as for mRNA expression level, as compared to MCF7 cells.

**Figure 4:**
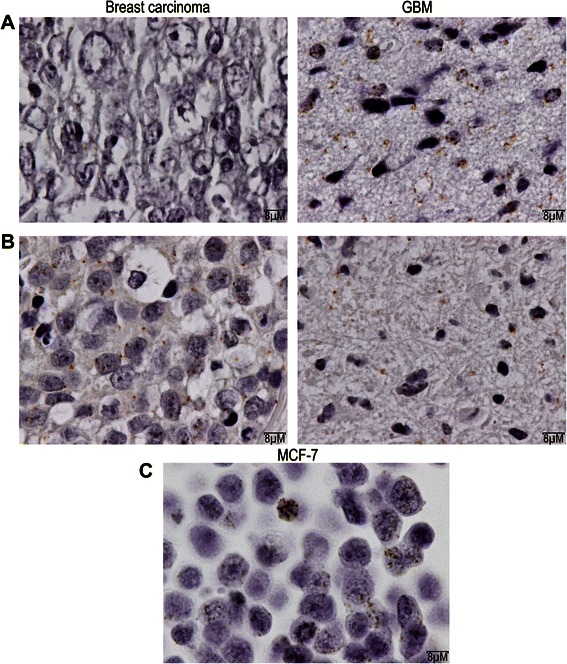
***BRCA1*protein expression by proximity ligation assay. A**. Patient 1. Left : breast tumour; Right : GBM. **B**. Patient 2. Left : breast tumour; Right: GBM. **C**. Positive control : MCF7 cells.

The protein expression level was 4,08% in breast tumour and 45,86 % in GBM of patient 1 and 19,04 % in breast tumour and 36,37% in GBM of patient 2. Representative images of the *BRCA1* protein expression in GBM of both patients are represented in Figure 4.

Promoter methylation, mRNA expression and protein expression levels of *BRCA1* are summarized in Table 1.

**Table 1:** **Summary of** ***BRCA1*** **molecular analysis**

	Breast carcinoma			Glioblastoma		
	*BRCA1*promoter methylation	*BRCA1*mRNA (%MCF7)	*BRCA1*protein (%MCF7)	*BRCA1*promoter methylation	*BRCA1*mRNA (%MCF7)	*BRCA1*protein (%MCF7)
Patient 1	Yes	18	4	No	8	46
Patient 2	No	10	19	No	13	36

## Discussion

Here, we report the cases of two patients, with a germline *BRCA1* mutation, who developed two primary breast cancers and a GBM. Both women present a germline pathogenic heterozygote mutation in the exon 11 of the *BRCA1* gene, leading to a truncated transcript.

In 80% of *BRCA1* breast carcinoma the protein expression is lost because of the deletion of the second allele [15,16]. Few cases of glioblastoma in *BRCA1* mutation carriers were reported but the *BRCA1* expression status has never been studied.

In an attempt to clarify the role played by a *BRCA1* mutation in GBM development, we performed diverse molecular experiments to characterize the expression status of *BRCA1* in glioblastoma, and in the first TNBC of the two patients.

Our data show that the *BRCA1* protein expression is maintained in glioblastoma suggesting that no loss of heterozygosity occurred in these tumours. The sequencing data of the tumoural *BRCA1* gene in the GBM of patient 2 support this hypothesis. The protein expression is tenfold higher in the glioblastoma of patient 1 than in her breast carcinoma, and twice higher in patient 2. However, the *BRCA1* expression level was never completely lost, even in the TNBC. This observation is in concordance with what is observed in breast cancer cell lines where *BRCA1* is mutated (HCC1937 ) or its promoter methylated (UACC3199) [17,18].

*BRCA1* expression is also known to be lost in some sporadic breast cancers after methylation of the gene promoter (13% of cases) [12,19], but promoter methylation is rarely observed in tumours of *BRCA1* mutation carriers [20]. However, we observed *BRCA1* promoter methylation in the triple negative breast carcinoma of patient 1, but not in glioblastoma. In agreement with the high protein expression level in the GBM, patient 2 did not present any *BRCA1* promoter methylation in this tumour. Therefore, in these two cases, despite of a *BRCA1* pathogenic germline mutation, the tumour-suppressor protein expression is maintained in GBM, suggesting that the *BRCA1* mutation is not instrumental for GBM development. This observation is consistent with cancer statistics that have not highlighted any increased risk for brain tumour development in *BRCA1* carriers [21,22]. However, these studies were conducted in 1994 and 2002. Since then, treatment of breast cancer has evolved and survival increased, maybe allowing the detection of a previously hidden link between mutation of *BRCA1* and the risk to develop GBM. Indeed, as breast cancer appears before GBM in BRCA1 mutated patients, increased survival because of improved treatments may now allow the recording of brain tumour development. Moreover, the work of Konishi and co-authors has demonstrated that a heterozygous mutation of *BRCA1* without loss of the wild type allele can still induce genome instability [23]. Thus, conserved *BRCA1* protein expression in glioblastoma does not completely rule out its role in GBM development.

Another way to establish a relation between the *BRCA1* mutation and the GBM development would be to estimate the difference between the observed incidence of cases and the theoretical occurrence risk of both events. In our European population and during the last 10 years of *BRCA1* mutation screening, approximately between 2 and 8 cases associating *BRCA1* mutation and GBM for 100 million women are expected [24-26], but only three cases have been reported in the literature (two in Europe in the same centre and one in United States). Moreover, we are not aware of any case of GBM in males with *BRCA1* mutation.

## Conclusion

Our study failed to establish any biological link between GBM and *BRCA1* mutation but further genotype/phenotype studies might be needed to finally demonstrate or exclude any relation. It is possible to imagine that phenotype specificities could be linked with an increased risk of unusual cancers in *BRCA1* mutated patients. Indeed our two patients had mutations generating truncated proteins. Moreover, they both developed bilateral breast cancers. Large studies are required to verify whether complete loss of function *BRCA1* mutations or interactions with other genes could be specifically associated with increased risk of rare cancers.

## Consent

Written informed consent was obtained from one direct relative of each patient for publication of these case reports and accompanying images.

A copy of the written consent is available for review by the Editor of this journal.
